# Early postoperative beta-blockers are associated with improved cardiac output after late complete repair of tetralogy of Fallot: a retrospective cohort study

**DOI:** 10.1007/s00431-024-05597-1

**Published:** 2024-05-09

**Authors:** Guillaume Maitre, Damien Schaffner, Sebastiano A. G. Lava, Marie-Hélène Perez, Stefano Di Bernardo

**Affiliations:** 1 Pediatric Intensive Care Unit, Women Mother and Child Department, Lausanne University Hospital, Lausanne, Switzerland; 2 Pediatric Cardiology Unit, Women Mother and Child Department, Lausanne University Hospital, Lausanne, Switzerland

**Keywords:** Cardiac output, low, Tetralogy of Fallot, Adrenergic beta-antagonists, Heart failure, diastolic, Heart defects, congenital

## Abstract

Tetralogy of Fallot is the most common cyanotic congenital heart disease. For decades, our institution has cared for humanitarian patients with late presentation of tetralogy of Fallot. They are characterized by severe right ventricular hypertrophy with consecutive diastolic dysfunction, increasing the risk of postoperative low cardiac output syndrome (LCOS). By right ventricular restrictive physiology, we hypothesized that patients receiving early postoperative beta-blockers (within 48 h after cardiopulmonary bypass) may have better diastolic function and cardiac output. This is a retrospective cohort study in a single-center tertiary pediatric intensive care unit. We included > 1-year-old humanitarian patients with a confirmed diagnosis of tetralogy of Fallot undergoing a complete surgical repair between 2005 and 2019. We measured demographic data, preoperative echocardiographic and cardiac catheterization measures, postoperative mean heart rate, vasoactive-inotropic scores, LCOS scores, length of stay, and mechanical ventilation duration. One hundred sixty-five patients met the inclusion criteria. Fifty-nine patients (36%) received early postoperative beta-blockers, associated with a lower mean heart rate, higher vasoactive-inotropic scores, and lower LCOS scores during the first 48 h following cardiopulmonary bypass. There was no significant difference in lengths of stay and ventilation.

*Conclusion*: Early postoperative beta-blockers lower the prevalence of postoperative LCOS at the expense of a higher need for vasoactive drugs without any consequence on length of stay and ventilation duration. This approach may benefit the specific population of children undergoing a late complete repair of tetralogy of Fallot.

**Table:** 

**What is Known:** *• Prevalence of low cardiac output syndrome is high following a late complete surgical repair of tetralogy of Fallot.*	
**What is New:** *• Early postoperative beta-blockade is associated with lower heart rate, prolonged relaxation time, and lower prevalence of low cardiac output syndrome.* *• Negative chronotropic agents like beta-blockers may benefit selected patients undergoing a late complete repair of tetralogy of Fallot, who are numerous in low-income countries.*

**Supplementary Information:**

The online version contains supplementary material available at 10.1007/s00431-024-05597-1.

## Introduction

In low-income countries, surgical repair of tetralogy of Fallot (ToF) is not always possible, and humanitarian charities refer children to hospitals that offer this kind of surgery. We recently described our cohort’s surgical outcomes of late complete repair of ToF [1]. Because of inherent diagnostic and logistical delays, these children are virtually always older than 1 year old when admitted for surgery. Consequently, they tend to have already developed a relevant right ventricular hypertrophy (RVH), putting them at high risk of postoperative low cardiac output syndrome (LCOS). The severity of RVH and associated restrictive physiology is related to the duration of pressure overload, which, in turn, is correlated with patients’ age and severity of pulmonary stenosis (PS) [2, 3]. Long-lasting RVH and hypoxia (leading to myocardial fibrosis) may be responsible for restrictive right ventricle (RV) physiology, which was demonstrated in patients undergoing ToF correction as soon as at 6 months of age [4, 5]. Looking at the early postoperative course in 50 patients with ToF, Sachdev et al. showed diastolic dysfunction in half of them with a significant association between restrictive RV physiology, prolonged stay, longer duration of inotropic support, and increased needs for diuretics [5]. Restrictive physiology manifests itself through diastolic dysfunction with higher filling pressures consecutive to (1) slowed or incomplete relaxation, (2) reduced ventricular filling, and (3) altered passive elastic properties due to collagen accumulation or altered collagen architecture [6]. Diastolic function plays a significant role in cardiac output [7]. Patients with RV restrictive physiology are at higher risk of postoperative LCOS despite excellent systolic function. The postoperative RV diastolic function is affected by the cardiopulmonary bypass (CPB) duration, myocardial edema, potential ventriculotomy, myocardial infundibular resection, and patches used to close the ventricular septal defect or enlarge the right ventricular outflow tract (RVOT) [4]. Propranolol, a nonselective beta-blocker (b-B), reduces RV hypercontractility, increases relaxation time and peripheral vascular resistance, and allows better pulmonary blood flow [8–10]. It has been used since the 1960s to prevent cyanotic spells [11–14]. Furthermore, an effect of propranolol on cardiomyocyte proliferation was recently described [15–17]. Its use was also described in adult patients with RV dysfunction following ToF repair [18].

The place of negative chronotropic agents in preventing LCOS after late complete ToF repair is unknown. In the context of pre-existing RV restrictive physiology and diastolic dysfunction, we hypothesized that early postoperative b-B administration may foster diastolic function and, thus, cardiac output unless it is contraindicated by severe pulmonary regurgitation (PR) or atrioventricular block, for instance. The objective of our study was to compare postoperative outcomes with the early postoperative use of b-B after late complete surgical ToF repair.

## Methods

This is an observational retrospective cohort study. It was conducted at the Lausanne University Hospital, a tertiary care teaching center performing approximately 150 to 200 congenital open-heart surgeries annually. Ethical approval was obtained from the local ethical board in December 2019 (ID number 2019–01701). Consent for study inclusion was waived.

Eligible for inclusion was > 1-year-old patients referred to our institution by humanitarian associations for a complete ToF surgical repair between January 2005 and January 2019. Children under 12 months at surgical repair, undergoing a palliative procedure, or patients with extracardiac malformations requiring additional surgical interventions during the CPB were excluded. We retrospectively collected the clinical information from the hospital’s electronic medical records (Soarian, Siemens^®^ and MetaVisionSuite, iMDSoft^®^). This included demographic information and anatomic cardiac diagnoses (including preoperative cardiac catheterization and transthoracic echocardiogram) at baseline. In addition, a pediatric cardiologist retrospectively measured the cardiac anatomic structures on transthoracic echocardiograms (Xcelera, Philips^®^). The following preoperative measures were collected: pulmonary valve diameter and Z-score, transpulmonary valve gradient, RVOT diameter, RV anterior wall thickness. In the absence of published right ventricular free wall thickness Z-scores, we expressed the degree of RVH by using the RV/LV ratio representing the ratio between RV anterior wall thickness and the left ventricle posterior wall thickness in diastole. This ratio is between 0.3 and 0.34 for healthy adults, whilst an RV/LV ratio > 0.8 represents significant RVH [19]. More directly correlated with RV diastolic function, the filling pressures of the right heart (mean right atrial pressure, RAP, and right ventricular end-diastolic pressure, RVEDP) were measured during preoperative cardiac catheterization. Missing data were excluded from the analyses.

We compared the following outcomes in relation to the postoperative administration of b-B within 48 h after CPB: length of pediatric intensive care unit (PICU) stay (number of days between PICU admission and PICU discharge), length of hospital stay (number of days between hospital admission and hospital discharge), total ventilation duration (hours of invasive ventilation, IV, and non-invasive ventilation, NIV). Mortality until hospital discharge and use of mechanical support were recorded. Mean heart rate and mean central venous pressure (CVP) were collected. Vaosactive-inotropic score (VIS) and prevalence of LCOS were calculated within 6-h intervals until 48 h after weaning from CPB. VIS was calculated as follows: dopamine (mg/kg/min) + dobutamine (mg/kg/ min) + [100 × adrenaline (mg/kg/min)] + [10 × milrinone (mg/kg/min)] + [10,000 × vasopressin (U/kg/ min)] + [100 × noradrenaline (mg/kg/min)] [20]. Invasive and continuous cardiac output monitoring is not routinely performed in the pediatric population; healthcare providers rely on clinical evaluation and indirect parameters of low cardiac output [21]. Therefore, we retrospectively defined LCOS with a composite score adapted from previously used LCOS criteria (Table 1) [22–24]. LCOS score was created as follows: lactatemia: 0 point if < 2 mmol/L, 1 point if 2–4 mmol/L, 2 points if > 4 mmol/L; D_av_O_2_ (= S_a_O_2_–S_cv_O_2_): 0 point if < 25%, 1 point if 25–35%, 2 points if > 35%); D_av_CO_2_ (= P_v_CO_2_–P_a_CO_2_): 0 point if < 6 mmHg, 1 point if 6–10 mmHg, 2 points if > 10 mmHg; urine output: 0 point if > 1 mL/kg/h, 1 point if 0.5–1 mL/kg/h, 2 points if < 0.5 mL/kg/h. No point was assigned in case of a missing variable. LCOS was defined as a LCOS score ≥ 6 points. To compare VIS and LCOS prevalence, a patient on b-B was defined as receiving b-B at the time of the post-CPB interval of interest.

**Table 1:** Demographic data

	**All patients (*n* = 165)**	**Within 48 postoperative hours**		
		**No beta-blockers** (*n* = 106, 64%)	**Beta-blockers** (*n* = 59, 36%)	***p* value**
**Sex male** (*n* = 165)	105 (64%)	72 (68%)	33 (56%)	0.13
**Age (years)** (*n* = 165)	4.5 [3.0; 6.3]	4.5 [3.0; 6.3]	4.3 [2.8; 6.0]	0.89
**Weight (kg)** (*n* = 165)	13.5 [10.9; 16.5]	13.5 [10.9; 16.1]	14 [10.9; 17.0]	0.61
**Weight (Z-score)** (*n* = 165)	−1.7 [−2.5; −1.0]	−1.8 [−2.6; −1.1]	−1.7 [−2.5; −0.8]	0.52
**Height (cm)** (*n* = 165)	98 [88; 112]	97 [87; 111]	99 [88; 114]	0.65
**Height (Z-score)** (*n* = 165)	−1.35 [−2.4; −0.7]	−1.5 [−2.5; −0.7]	−1.2 [−2.2; −0.5]	0.11
**BMI (kg/m**^**2**^**)** (*n* = 165)	14.0 [12.8; 15.2]	14.1 [12.9; 15.3]	13.8 [12.7; 15]	0.51
**BMI (Z-score)** (*n* = 165)	−1.3 [−2.3; −0.3]	−1.3 [−2.3; −0.3]	−1.3 [−2.5; −0.4]	0.56
**BSA (m**^**2**^**)** (*n* = 165)	0.6 [0.5; 0.7]	0.6 [0.5; 0.7]	0.6 [0.5; 0.8]	0.62
**Baseline SpO**_**2**_ (%) (*n* = 148)	78 [70; 85]	79 [71; 86]	77 [70; 81]	0.09
**Baseline Hb (g/L)** (*n* = 158)	163 [142; 190]	140 [101; 163]	163 [144; 196]	0.54
**Baseline Hct (%)** (*n* = 158)	50.5 [44; 59]	50 [44; 61]	51 [45; 58]	0.82

Demographics data displayed for all patients and subgroups in relation to early postoperative use of beta-blockers. Discrete variables are displayed as numbers (proportion), and continuous variables are displayed as median [interquartile range]. Missing data are displayed in the first column. Significance is inferred with a *p* value < 0.05 and identified with a *

*BMI* body mass index, *BSA* body surface area, *Hb* hemoglobin, *Hct* hematocrit, *IHA* initial health assessment, SpO_2_ pulsatile oxygen saturation

Statistical analysis was performed with Stata 17.0 (StatCorp^®^). Proportions are presented as absolute numbers and percentages and were analyzed with the Chi-square test or Fisher’s exact test, as appropriate. Continuous variables are presented as median and [interquartile range] and were compared with the Wilcoxon-Mann–Whitney test, respectively, the Kruskal–Wallis one-way ANOVA test. Relationships between continuous variables were assessed with the Pearson correlation test, continuous and ordinal variables with Kendall’s Tau test, and continuous and categorical variables with the Point-Biserial correlation test. Statistical significance was inferred at a value of *p* < 0.05.

## Results

One hundred sixty-nine patients met the inclusion criteria. Four were excluded because of extracardiac malformations (severe tracheal stenosis). There was no significant difference in demographic data and baseline characteristics (like anthropometric measures or nutritional status [25]) between patients who received early postoperative b-B and those who did not (Table 1). There was a significant increase in patients receiving b-B in the early postoperative period over the years: while no patient received early postoperative b-B between 2005 and 2010, and > 50% was treated with b-B between 2014 and 2018.

**Fig. 1:**
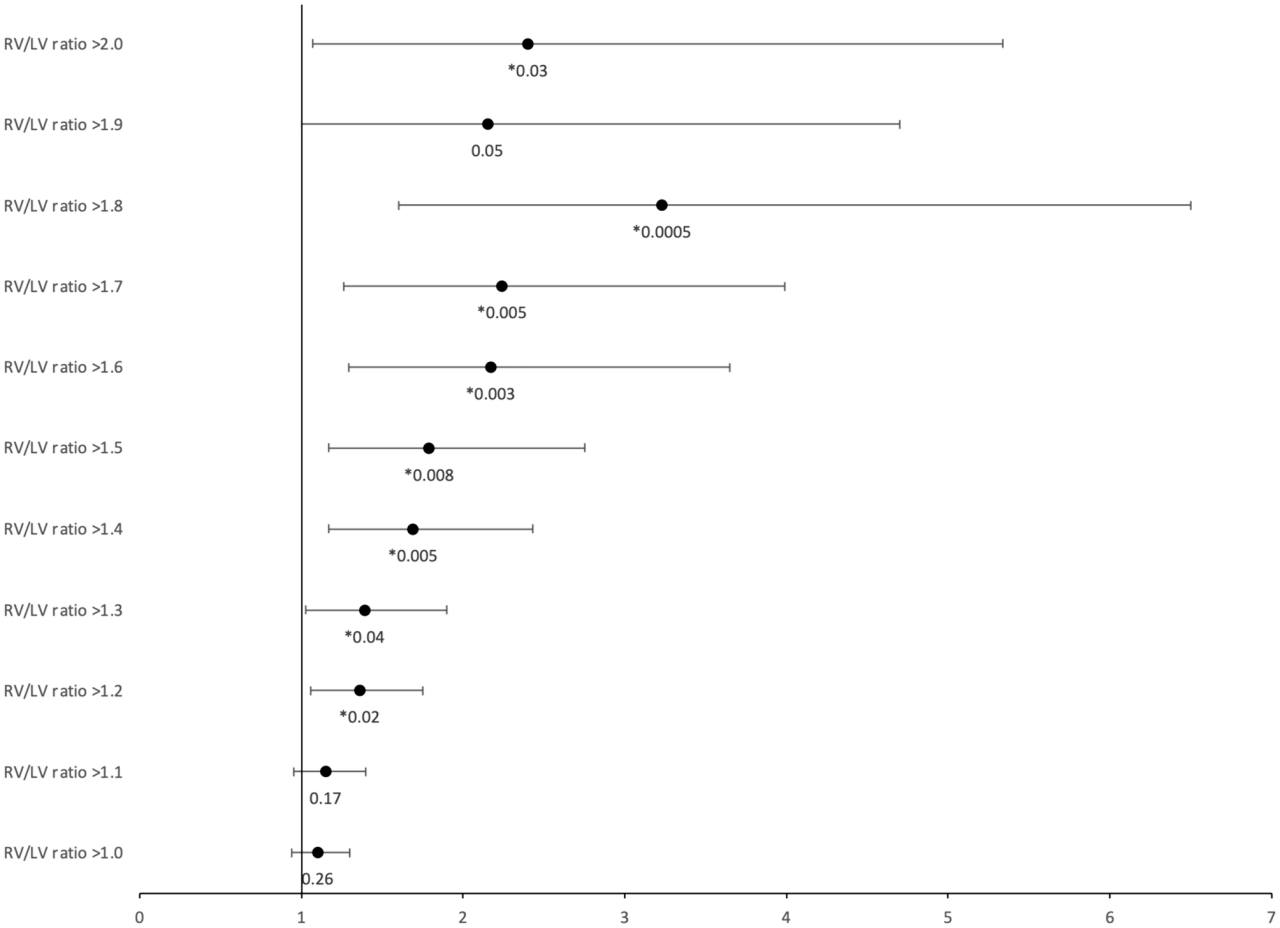
Risk ratios for use of early postoperative beta-blockers according to RV/LV ratio. 95% confidence intervals and *p* values are displayed in the graph, * indicates significance inferred with a *p* value < 0.05. RV/LV ratio: ratio between right ventricle anterior wall thickness and left ventricle posterior wall thickness in diastole

All treated patients received oral propranolol with a median unitary dose of 0.6 mg/kg [0.4;0.9] and a maximal dose of 0.8 mg/kg, three to four times a day 0.8 mg/kg [0.5;1], three to four times a day. For a minority of them (13.5%), intravenous esmolol was initially used with a median dose of 45 mg/kg/min [26.7;68.9] and a maximal dose of 90 mg/kg/min [38.4; 445.5]. The timing of initiation of b-B, either intravenous or enteral, was 5.3 h after CPB [3.8; 21.5]. There was no significant correlation between the time of initiation and preoperative echocardiographic measures of RVH and PS or the right-heart filling pressures (RAP and RVEDP). Neither the use of early postoperative b-B nor the propranolol dose was significantly associated with the preoperative echocardiographic and cardiac catheterization measures (eTable 1), with the notable exception that patients with an RV/LV ratio > 1.2 were more likely to receive early postoperative b-B (RR 1.4 [1.1; 1.8], *p* = 0.02, Fig. 1). The median of mean CVP was between 10.9 and 12 mmHg for every postoperative 6-h interval. There was no significant difference in mean CVP between the two subgroups (eTable 2).

The proportions of patients receiving postoperative b-B differed significantly according to the type of surgical correction. Three out of 25 (12%) patients with the insertion of a valved conduit received postoperative b-B. At the same time, this was the case for 30/63 (48%) and 26/77 (34%) of patients with transpulmonary patch (TP) and pulmonary valve-sparing repair (PVSR), respectively (*p* = 0.006). Conversely, more than half of the patients treated with early postoperative b-B had undergone a repair through TP (eFigure 1). The timing of initiation of b-B did not significantly differ as per the chosen surgical intervention: 5.1 h [3.5;21.2] for TP, 10.6 [4.8;23.0] for PVSR, and 4.0 [4.0;5.0] for RV-PA valved conduit insertion (*p* = 0.09). The duration of CPB did not significantly differ between patients having received postoperative b-B and those who did not (139 min [112;170] vs. 121.5 min [105;149], *p* = 0.06). Similarly, there was no correlation between the duration of CPB and the timing of initiation of b-B (*p* = 0.39). However, patients undergoing a TP had a significantly longer CPB compared with the two other surgical strategies (147 min [118–173] vs. 114 [94–135] for PVSR and 130 [113–146] for RV-PA, *p* = 0.0001).

**Fig. 2:**
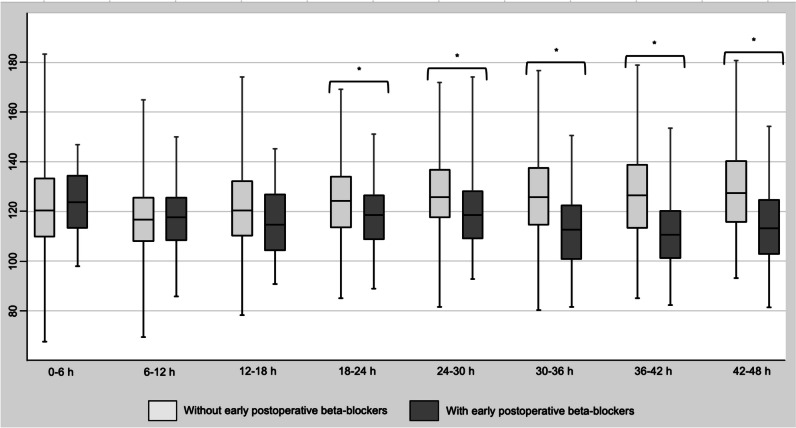
Mean heart rate by 6-h interval after CPB. Mean value, interquartile range, and minimal and maximal values are represented in the graph. Significance is inferred with a *p* value < 0.05 and identified with a *

There was no significant difference in the length of PICU and hospital stay, invasive, non-invasive, and total ventilation durations between patients receiving early postoperative b-B or not (Table 2). No mortality or mechanical support was encountered in our cohort. The use of beta-blockers within the first 48 postoperative hours was associated with a significantly lower mean heart rate from the 18–24 h post-CPB interval. This difference was significant for several time intervals (Fig. 2). VIS was significantly higher for those receiving early postoperative b-B from 6 to 12 h post-CPB (Table 2). There was no significant association between VIS and the occurrence of LCOS, except for the interval between 36 and 42 h post-CPB for patients receiving b-B (eTable 3).

**Table 2:** Comparison of patient outcomes with early postoperative beta-blockers use and Vasoactive-Inotropic Scores (VIS) within 48 h following CPB. Continuous variables are displayed as median [interquartile range]. Missing data are displayed in the table. Significance is inferred with a *p* value < 0.05 and identified with a *

	**No early postoperative b-B**	**Early postoperative b-B**	***p* value**
**Length of PICU stay (days)**	6.0 [4.7; 7.7] *n* = 106	6.1 [4.7;8.1] *n* = 59	0.7
**Length of hospital stay (days)**	10 [8; 14] *n* = 106	8 [7;12] *n* = 59	0.13
**Invasive ventilation duration (hours)**	25.7 [22.0;69.8] *n* = 99	43.5 [19.0;76.5] *n* = 55	0.85
**Non-invasive ventilation duration (hours)**	19.1 [11.0;24.2] *n* = 28	16.1 [10.3;22] *n* = 25	0.53
**Total ventilation duration (hours)**	28.3 [22.8;71.8] *n* = 99	44.7 [22.5;91.4] *n* = 55	0.46
**VIS—0–6 h post CPB**	26.1 [19.0; 50.0] *n* = *126*	40.5 [20.5; 55.6] *n* = *30*	0.07
**VIS—6–12 h post CPB**	25.7 [12.8; 37.3] *n* = *120*	34.5 [19.0; 53.0] *n* = *36*	0.02*
**VIS—12–18 h post CPB**	29.3 [14.6; 44.5] *n* = *119*	40.9 [25.6; 60.5] *n* = *37*	0.01*
**VIS—18–24 h post CPB**	29.8 [14.6; 44.8] *n* = *108*	38.6 [21.1; 57.8] *n* = *48*	0.02*
**VIS—24–30 h post CPB**	27.1 [12.4; 41.7] *n* = *102*	40.9 [21; 52.6] *n* = *54*	0.01*
**VIS—30–36 h post CPB**	27.7 [11.5; 40.8] *n* = *102*	38.9 [21.9; 51.5] *n* = *54*	0.01*
**VIS—36–42 h post CPB**	28.4 [12.4; 41.8] *n* = *102*	38.5 [22; 53.4] *n* = *54*	0.01*
**VIS—42–48 h post CPB**	29 [12.4; 41.4] *n* = *99*	40.2 [22; 53.4] *n* = *57*	0.01*

*b-B* beta-blockers, *CPB* cardiopulmonary bypass

**Fig. 3:**
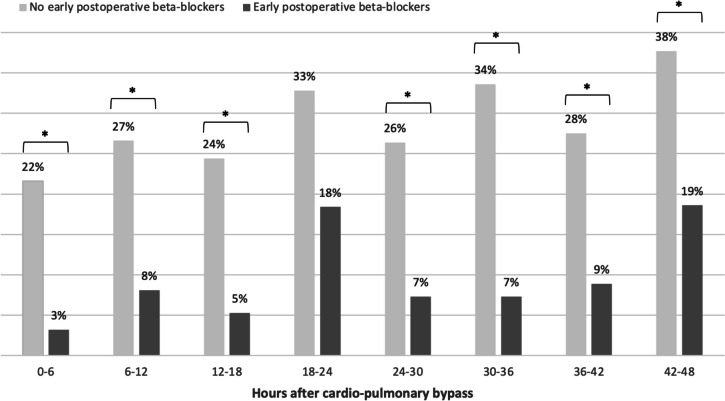
LCOS prevalence by time intervals after CPB. Comparison of LCOS prevalence according to the use of early postoperative b-B. All surgical strategies are included. Significance is inferred with a *p* value < 0.05 and identified with a *

At every 6-h interval, the LCOS prevalence was lower in the group treated with postoperative b-B, ranging from 1/31 (3%) to 9/49 (18%) vs. 29/134 (22%) to 37/110 (38%) in the non-treated group (Fig. 3). These differences were significant except for the 18–24 h post-CPB interval. Looking at subgroups by surgical strategy, the significant differences were essentially for patients undergoing a complete repair with a TP (eTable 4).

## Discussion

At our institution, the practice of early postoperative b-B with propranolol after TOF repair was started in 2011 and quickly became a common practice, with more than 50% of patients treated between 2014 and 2019. This proportion of postoperative b-B is much higher than previously reported in a North American national retrospective database review [26], in which 10% of admitted patients received postoperative b-B. However, this study’s population was not comparable with ours. Their use was based on individual intensivist’s preferences according to the patient’s clinical situation and evolution during the first postoperative hours. Pre-existing or new heart block was a contraindication to b-B administration. From a theoretical framework, the diastolic function is correlated with relaxation time; with a lower heart rate, more time is spent in diastole, and ventricular filling capacity is enhanced. This concept was also well described in adults presenting heart failure with preserved left ventricular ejection fraction [27]. Indeed, in our cohort, early postoperative b-B significantly decreased the mean heart rate from 18 h after CPB. This negative chronotropic effect lasted until at least 48 h after CPB. This means that the clinical goal of increasing relaxation time was achieved, with an assumptive improvement of their diastolic function. Many clinical aspects (peripheral perfusion, perfusion pressure, postoperative echocardiogram) may play a role in selecting patients started on early postoperative b-B; we could not point those out. However, our retrospective analysis showed that patients with a higher preoperative RV/LV ratio were more likely to receive early postoperative b-B. A thicker RV predisposes to diastolic dysfunction, and we hypothesized that this may have prompted clinicians to use early postoperative b-B.

We described the various surgical strategies for ToF repair and their determinants in a previous publication [1]. The low proportion of patients undergoing a PVSR may be explained by the poor quality of the pulmonary valve after years of unrepaired ToF. The humanitarian nature of the surgical management may also prompt the surgeon to opt for a definitive procedure with less risk for reoperation. In our cohort, patients undergoing a repair with TP were more likely to receive early postoperative b-B. We showed in a previous descriptive study on the same cohort that patients undergoing a repair with TP had a higher RVOT gradient, smaller pulmonary valve annulus, and greater RV hypertrophy [1]. These significative differences in the preoperative echocardiographic assessment may prompt the clinician to use early postoperative beta-blockers. Due to diastolic dysfunction with high filling pressures, the expected degree of PR consecutive to a TP may not be relevant in the early postoperative course, so this surgical strategy, as such, does not prohibit the use of b-B during the initial postoperative period. Consequently, patients undergoing a repair with TP also presented a significantly lower prevalence of postoperative LCOS. Their longer CPB duration than other surgical strategies may partly explain the more frequent use of b-B in this patient group. Although we did not identify significant differences in preoperative markers of RVH, PS, and filling pressures, a more severe obstruction of the RVOT not allowing the surgeon to spare the pulmonary valve could also have been contributive.

This study highlights that early postoperative b-B after complete repair of ToF is associated with a lower prevalence of LCOS at the expense of more vasoactive support. However, the occurrence of LCOS was not associated with a lower vasoactive-inotropic score. We interpret the significant association between early postoperative b-B and lower LCOS prevalence as a sign of improved diastolic function in the treated cohort. Indeed, rather than waiting for signs of a good cardiac output before starting b-B, whose indication would be questionable at that time point, we hypothesize that (in this retrospective observational study) clinicians were tempted to start b-B early with the aim to enhance the diastolic function during the critical early postoperative period. The presented data supports the notion that early postoperative b-B is beneficial in minimizing or preventing LCOS in this setting. A greater need for vasoactive drugs following the administration of b-B is anticipated because of the inhibitory effect on sympathetic activation. The higher VIS scores than usually reported in similar contexts are explained by the previously described strategy followed at our unit using milrinone and noradrenaline instead of adrenaline in the postoperative management [28]. Despite a greater need for vasoactive drugs, patients on early postoperative b-B experience the same postoperative course as those who were not treated. There were no significant differences in the length of PICU, hospital stay, or ventilation durations. The older age of our cohort partially explains the relatively short ventilation durations. While comparing the PICU length of stay across several studies, logistical factors related to our internal institutional organization and the step-down unit’s capacity should be considered as relevant modulators of this outcome measure.

The retrospective nature of our study confers various limitations. First, early postoperative assessment was based on inotropic score, while clinical and echocardiographic data were unavailable. For instance, presence or absence of hepatomegaly and hepatojugular reflux, jugular vein assessment, echocardiographic measures of diastolic function, quantification of PR, and residual dynamic PS would have provided additional insights. Mean CVP measurements were high in every postoperative 6-h interval for all patients and may represent an indirect sign of diastolic dysfunction in the absence of objective assessment. Similarly, markers of end-organ impairment would have allowed a more comprehensive analysis. Second, over 13 years, many changes may have occurred (change of cardiac surgeons, evolution of preoperative assessment, surgical techniques, and postoperative management) [29]. For instance, sedation strategies evolved with the uptake of alpha-agonist agents (clonidine, dexmedetomidine) with a negative chronotropic effect confounding the effect of b-B. We were not able to adjust for these confounding factors. Moreover, it should be acknowledged that despite a careful and restrictive scoring system, the retrospective definition of LCOS, albeit frequently used in pediatric studies [22–24], and probably the best research tool currently available to measure its occurrence, still suffers from some imprecision. Finally, longer-term outcomes (like functional capacity, for instance [30]) were not assessed.

The results of this study must be interpreted with an understanding of the peculiarities of the included population, which preclude any generalizability to other pediatric populations. In particular, we would not recommend early postoperative b-B for neonates and infants undergoing a ToF repair, as they do not present the same degree of RVH and fibrosis as our cohort, and, most importantly, they are more exposed to negative inotropism.

To the best of our knowledge, this is the first descriptive study about the postoperative use of b-B in a population of children with late surgical repair of ToF. Furthermore, this cohort is at the same time also one of the biggest ever described with this condition [31]. This population’s representatives are numerous in developing countries. This study brings unprecedented data that informs some knowledge gaps surrounding the medical management of this unique population and may guide both clinical care and further research. We explored potential factors leading the caring physicians to prescribe postoperative b-B. However, further efforts should aim at identifying objective criteria to guide postoperative therapy and anticipate the patients who may benefit from this treatment strategy.

## Conclusion

Over the last decade, there has been a growing interest in diastolic function assessment and support in adult and pediatric cardiology. Besides the positive effect of propranolol on the occurrence of cyanotic spells, a pivotal role in reducing mortality and morbidity by protecting the chronically failing myocardium against hyperadrenegy (neurohormonal model of heart failure), and likely on cardiomyocyte division and proliferation, the role of beta-blockade on diastolic function in the early postoperative period following late surgical ToF repair stimulates renewed attention.

### Supplementary Information

Below is the link to the electronic supplementary material.

Supplementary file1 (DOCX 52 KB)

## Data Availability

No datasets were generated or analysed during the current study.

## Abbreviations

b-B: Beta-blockers
CPB: Cardiopulmonary bypass
CVP: Central venous pressure
LCOS: Low cardiac output syndrome
PICU: Pediatric intensive care unit
PR: Pulmonary regurgitation
PS: Pulmonary stenosis
PVSR: Pulmonary valve-sparing repair
RAP: Right atrial pressure
RV: Right ventricle
RVEDP: Right ventricular end-diastolic pressure
RVH: Right ventricular hypertrophy
RVOT: Right ventricular outflow tract
ToF: Tetralogy of Fallot
TP: Transpulmonary patch
VIS: Vasoactive inotropic score
