# Supplementary material for: Early postoperative beta-blockers are associated with improved cardiac output after late complete repair of tetralogy of Fallot: a retrospective cohort study
Source: Eur J Pediatr. 2024 May 9;183(8):3309–17. doi: 10.1007/s00431-024-05597-1 (PMC11263431; doi:10.1007/s00431-024-05597-1)
Supplement: Supplementary file 1 — Supplementary file1 (DOCX 52 KB) [file 431_2024_5597_MOESM1_ESM.docx]

**Early postoperative beta-blockers are associated with improved cardiac output after late complete repair of tetralogy of Fallot: a retrospective cohort study.**

Guillaume Maitre^1^, Damien Schaffner^2^, Sebastiano A.G. Lava^2^, Marie-Hélène Perez^1^, Stefano Di Bernardo^2^

**SUPPLEMENTAL MATERIAL**

Page 2 : eFigure 1 - **Distribution of included patients among treatment groups and surgical strategies.**

Pages 3-5 : eTable 1 - **Echocardiographic and cardiac catheterization data.**

Page 6 : eTable 2 - **Mean central venous pressure measurement in the postoperative period by 6-hour intervals.**

Page 7 : eTable 3 - **Vasoactive-Inotropic score and LCOS by postoperative 6-hour intervals**

Pages 8-9 : eTable 4 - **Incidence of LCOS within 48 postoperative hours by 6-hour time intervals and type of surgical repair.**

*p=0.006

**eFigure 1: Distribution of included patients among treatment groups and surgical strategies**. Numbers, proportions, and distribution of patients regarding the use of early postoperative beta-blockers and the surgical repair strategy for tetralogy of Fallot. Significance is interfered with a p-value <0.05 and identified with an *. *b-B: beta-blockers, PVSR: pulmonary valve sparing repair, RV-PA: repair with a right ventricle to pulmonary artery valved conduit, ToF: Tetralogy of Fallot, TP: repair with transpulmonary patch.*

**eTable 1: Echocardiographic and cardiac catheterization data.** Significance is inferred with a p-value < 0.05 and identified with a *.

*IHA: Initial Health Assessment, LV: left ventricle, RAP: Right atrium mean pressure, RV: right ventricle, RVEDP: right ventricle end-diastolic pressure, RVOT: Right ventricular outflow tract, TPV: trans pulmonary valve.*

|  | **All patients (n=165)** | **Within 48 postoperative hours** | | |
| --- | --- | --- | --- | --- |
|  |  | **No Beta-blockers**  **(n=106, 64%)** | **Beta-blockers**  **(n=59, 36%)** | **p-value** |
| **Pulmonary valve diameter (cm) (n=156)** | | | | |
|  | **1.1 [0.87 ; 1.3]** | **1.1 [0.9 ; 1.3]** | **1.1 [0.9 ; 1.25]** | **0.42** |
| ≤ 0.5cm | 2 (1.3%) | 0 | 2 (3.6%) | 0.28 |
| 0.5-0.8 cm | 23 (14.7%) | 13 (13.0%) | 10 (17.9%) |  |
| 0.8-1.1 cm | 73(46.8%) | 46 (46.0%) | 27 (48.2%) |  |
| 1.1-1.4 cm | 38 (24.4%) | 27 (27.0%) | 11 (19.6%) |  |
| 1.4-1.7 cm | 14 (9%) | 11 (11.0%) | 3 (5.4%) |  |
| 1.7 – 2 cm | 3 (1.9%) | 1 (1.0%) | 2 (3.6%) |  |
| > 2 cm | 3 (1.9%) | 2 (2.0%) | 1 (1.8%) |  |
| **Pulmonary valve Z-score (n=156)** | | | | |
|  | **-1.8 [-3.4 ; -0.8]** | **-1.8 [-3.1 ; -0.7]** | **-2 [-3.5 ; -0.9]** | **0.32** |
| ≤ -6 | 4 (2.9%) | 1 (1.2%) | 3 (5.9%) | 0.5 |
| -6 - -4 | 20 (14.5%) | 12 (13.8%) | 8 (15.7%) |  |
| -4 - -2 | 46 (33.3%) | 29 (33.3%) | 17 (33.3%) |  |
| -2 – 0 | 67 (48.6%) | 44 (50.6%) | 23 (45.1%) |  |
| 0 – 2 | 0 | 0 | 0 |  |
| > 2 | 1 (0.7%) | 1 (1.2%) | 0 |  |
| **TPV gradient (mmHg) (n=149)** | | | | |
|  | **77 [62 ; 88]** | **77 [64 ; 90]** | **77.5 [56 ; 86]** | **0.29** |
| ≤ 20 mmHg | 2 (1.3%) | 1 (1.1%) | 1 (1.9%) | 0.28 |
| 20-40 mmHg | 6 (4.0%) | 4 (4.2%) | 2 (3.7%) |  |
| 40-60 mmHg | 28 (18.8%) | 13 (13.7%) | 15 (27.8%) |  |
| 60-80 mmHg | 52 (34.9%) | 38 (40%) | 14 (25.9%) |  |
| 80-100 mmHg | 47 (31.5%) | 28 (29.5%) | 19 (35.2%) |  |
| 100-120 mmHg | 9 (6.0%) | 7 (7.4%) | 2 (3.7%) |  |
| >120 mmHg | 5 (3.4%) | 4 (4.2%) | 1 (1.9%) |  |
|  | | | | |
|  | | | | |
| **RV anterior wall thickness (mm) (n=161)** | | | | |
|  | **6.0 [5.3 ; 6.9]** | **6 [5.3 ; 6.9]** | **6 [5.4 ; 6.9]** | **0.88** |
| ≤ 3mm | 1 (0.6%) | 0 | 1 (1.7%) | 0.37 |
| 3-5mm | 33 (20.5%) | 24 (23.5%) | 9 (15.3%) |  |
| 5-7mm | 92 (57.1%) | 57 (55.9%) | 35 (38%) |  |
| 7-9mm | 30 (18.6%) | 19 (18.6%) | 11 (18.6%) |  |
| 9-11mm | 3 (1.9%) | 2 (2%) | 1 (1.7%) |  |
| 11-13mm | 1 (0.6%) | 0 | 1 (1.7%) |  |
| >13mm | 1 (0.6%) | 0 | 1 (1.7%) |  |
| **RV/LV ratio (n=147)** | | | | |
|  | **1.2 [1.0 ; 1.5]** | **1.19 [1 ; 1.42]** | **1.3 [1.04 ; 1.63]** | **0.07** |
| ≤ 1.0 | 36 (24.5%) | 26 (26.3%) | 10 (20.8%) | 0.36 |
| 1.0-1.5 | 75 (51%) | 53 (53.5%) | 22 (45.8%) |  |
| 1.5-2.0 | 33 (22.5%) | 18 (18.2%) | 15 (31.3%) |  |
| > 2.0 | 3 (2.0%) | 2 (2.0%) | 1 (2.1%) |  |
| **RVOT diameter (mm) (n=115)** | | | | |
|  | **7.4 [5.8 ; 9.7]** | **7.45 [5.8 ; 9.7]** | **7.3 [6 ; 9]** | **0.61** |
| ≤ 4mm | 5 (4.4%) | 2 (2.7%) | 3 (7.3%) | 0.23 |
| 4-6mm | 29 (25.2%) | 21 (28.4%) | 8 (19.5%) |  |
| 6-8mm | 37 (32.2%) | 19 (25.7%) | 18 (43.9%) |  |
| 8-10mm | 25 (21.7%) | 19 (25.7%) | 6 (14.6%) |  |
| 10-12mm | 12 (10.4%) | 7 (9.5%) | 5 (12.2%) |  |
| 12-14mm | 5 (4.4%) | 4 (5.4%) | 1 (2.4%) |  |
| >14mm | 2 (1.7%) | 2 (2.7%) | 0 |  |
| **RVEDP (mmHg) (n=139)** | | | | |
|  | **8 [6 ; 10]** | **8 [6 ; 11]** | **7 [6 ; 10]** | **0.76** |
| ≤ 5 mmHg | 31 (22.3%) | 20 (22%) | 11 (22.9%) | 0.79 |
| 5-10 mmHg | 74 (53.2%) | 47 (51.7%) | 27 (56.3%) |  |
| 10-15 mmHg | 29 (20.9%) | 21 (23.1%) | 8 (16.7%) |  |
| 15-20 mmHg | 4 (2.9%) | 2 (2.2%) | 2 (4.2%) |  |
| 20-25 mmHg | 0 | 0 | 0 |  |
| > 25mmHg | 1 (0.7%) | 1 (1.1%) | 0 |  |
|  | | | | |
|  | | | | |
| **RAP (mmHg) (n=140)** | | | | |
|  | **7 [5 ; 9]** | **7 [5 ; 9]** | **7[5 ; 9]** | **0.39** |
| ≤5mmHg | 45 (32.1%) | 25 (27.2%) | 20 (41.7%) | 0.24 |
| 5-10 mmHg | 76 (54.3%) | 53(57.6%) | 23 (47.9%) |  |
| 10-15 mmHg | 16 (11.4%) | 11 (12%) | 5 (10.4%) |  |
| > 15 mmHg | 3 (2.1%) | 3 (3.3%) | 0 |  |

**eTable 2: Mean central venous pressure measurement in the postoperative period by 6-hour intervals.**

Median and [interquartile range] are displayed. Significance is inferred with a p-value < 0.05 and identified with a *.

|  | **All patients** | **No early postoperative b-B** | **Early postoperative b-B** | **p-value** |
| --- | --- | --- | --- | --- |
| H0-H6 (n=160) | 11.4 [9.2 ;14.3] | 11.8 [9.3 ;14.5] | 10.8 [9.1 ;12.5] | 0.11 |
| H6-H12 (n=160) | 10.9 [9.3 ; 13.1] | 11.1 [9.3 ;13.3] | 10.5 [9.4 ;12] | 0.53 |
| H12-H18 (n=160) | 12 [10.4 ;14.2] | 12 [10 ;14.3] | 12 [9.3 ;13.4] | 0.43 |
| H18-H24 (n=159) | 12.2 [10.4 ;14.2] | 12.7 [10.5 ;14.5] | 11.5 [10.3 ;13.2] | 0.16 |
| H24-H30 (n=159) | 12 [10.2 ;14.3] | 12.3 [10.2 ;14.3] | 11.5 [10.3 ;13.8] | 0.44 |
| H30-H36 (n=158) | 11.9 [10.1 ;13.8] | 11.8 [9.9 ; 14.3] | 12 [10.2 ;13.7] | 0.67 |
| H36-H42 (n=158) | 11.4 [8.9 ; 13.6] | 11.1 [8.5 ;13.7] | 11.8 [9.2 ;13.6] | 0.44 |
| H42-H48 (n=158) | 11.9 [93 ;13.9] | 11.8 [8.9 ;13.9] | 12 [10 ;13.9] | 0.44 |

**eTable 3: Vasoactive-Inotropic score and LCOS by postoperative 6-hour intervals.**

Median and [interquartile range] are displayed. Significance is inferred with a p-value < 0.05 and identified with a *.

| **Patients with early postoperative beta-blockers** | **No LCOS** | **LCOS** | **p-value** |
| --- | --- | --- | --- |
| H 0-6 | 40.5 [20.5;55.5] | 233 [233;233] | 0.07 |
| H 6-12 | 34.5 [20.5;50.3] | 41.6 [7.6;218.3] | 0.8 |
| H 12-18 | 39.2 [21.5;60.5] | 128.3 [40.9;215.6] | 0.3 |
| H 18-24 | 39.5 [21.5;58.2] | 29.6 [20.6;45.5] | 0.6 |
| H 24-30 | 41.4 [22.4;54.5] | 22.7 [3.8;45.2] | 0.2 |
| H 30-36 | 38.9 [22;55.7] | 33.8 [17.5;41.8] | 0.4 |
| H 36-42 | 42.7 [28.5;55.7] | 7.6 [7.5;21] | 0.01* |
| H 42-48 | 40.2 [21.5;57] | 37.8 [23.2;48.1] | 0.4 |

| **Patients without early postoperative beta-blockers** | **No LCOS no** | **LCOS** | **p-value** |
| --- | --- | --- | --- |
| H 0-6 | 27.2 [20;47.5] | 23.8 [11.9;56.5] | 0.9 |
| H 6-12 | 25.9 [14.6;37.3] | 25.2[7.5;37.3] | 0.7 |
| H 12-18 | 30 [16.2;43.6] | 20 [8;46] | 0.5 |
| H 18-24 | 31.4 [18.7;44.6] | 28.5 [10.2;45] | 0.5 |
| H 24-30 | 27.1 [14.6;44.8] | 24.4 [9.1;38] | 0.5 |
| H 30-36 | 28.4 [12.5;42.2] | 25.4 [9.9;38] | 0.4 |
| H 36-42 | 30 [15;41.8] | 27.8[10.2;38.8] | 0.5 |
| H 42-48 | 31 [15.7;44.6] | 27.5 [8;38.8] | 0.1 |

**eTable 4 : Incidence of LCOS within 48 postoperative hours by 6-hour time intervals and type of surgical repair.**

Comparison of LCOS incidence according to the use of early postoperative B-b. Significance is inferred with a p-value < 0.05 and identified with a *. *b-B: beta-blockers, CPB: cardiopulmonary bypass, PVSR : pulmonary valve sparing repair, RVPA: right ventricle to pulmonary artery*

| **Time intervals post CPB (hours)** | **Type of complete surgical repair of TOF** | **LCOS incidence without early postoperative b-B** | **LCOS incidence with early postoperative b-B** | **RR [95% CI]** | **p-value** |
| --- | --- | --- | --- | --- | --- |
| **0-6** | Transannular patch (*n=63)* | 11 (24.4%) | 1 (5.6%) | 0.23 [0.03 ; 1.63] | 0.085 |
|  | PVSR  *(n=77)* | 10 (14.9%) | 0 | 0 | 0.19 |
|  | RVPA conduit (*n=25)* | 8 (36.4%) | 0 | 0 | 0.2 |
| **6-12** | Transannular patch (*n=63)* | 12 (27.9%) | 2 (10%) | 0.36 [0.09 ; 1.45] | 0.11 |
|  | PVSR  *(n=77)* | 14 (22.2%) | 1 (7.1%) | 0.32 [0.05 ; 2.25] | 0.2 |
|  | RVPA conduit (*n=25)* | 8 (36.4%) | 0 | 0 | 0.2 |
| **12-18** | Transannular patch (*n=63)* | 12 (27.9%) | 1 (5%) | 0.18 [0.03 ; 1.3] | 0.04* |
|  | PVSR  *(n=77)* | 11 (17.7%) | 1 (6.7%) | 0.38 [0.05 ; 2.7] | 0.29 |
|  | RVPA conduit (*n=25)* | 8 (36.4%) | 0 | 0 | 0.2 |
| **18-24** | Transannular patch (*n=63)* | 13 (35.1%) | 4 (15.4%) | 0.44 [0.16 ; 1.2] | 0.08 |
|  | PVSR  *(n=77)* | 15 (26.3%) | 5 (25%) | 0.95 [0.4 ; 2.3] | 0.9 |
|  | RVPA conduit (*n=25)* | 10 (45.5%) | 0 | 0 | 0.13 |
| **24-30** | Transannular patch (*n=63)* | 11 (31.4%) | 1 (3.6%) | 0.1 [0.02 ; 0.83] | 0.005* |
|  | PVSR  *(n=77)* | 10 (18.9%) | 3 (12.5%) | 0.66 [0.2 ; 2.2] | 0.49 |
|  | RVPA conduit (*n=25)* | 8 (36.4%) | 0 | 0 | 0.2 |
| **30-36** | Transannular patch (*n=63)* | 13 (37.4%) | 1 (3.6%) | 0.1 [0.01 ; 0.7] | 0.001* |
|  | PVSR  *(n=77)* | 16 (30.2%) | 3 (12.5%) | 0.4 [0.13 ; 1.3] | 0.095 |
|  | RVPA conduit (*n=25)* | 8 (36.4%) | 0 | 0 | 0.2 |
| **36-42** | Transannular patch (*n=63)* | 11 (31.4%) | 1 (3.6%) | 0.11 [0.02 ; 0.83] | 0.005* |
|  | PVSR  *(n=77)* | 11 (21.2%) | 4 (16%) | 0.76 [0.27 ; 2.14] | 0.59 |
|  | RVPA conduit (*n=25)* | 8 (36.4%) | 0 | 0 | 0.2 |
| **42-48** | Transannular patch (*n=63)* | 16 (48.5%) | 4 (13.3%) | 0.28 [0.1 ; 0.73] | 0.003* |
|  | PVSR  *(n=77)* | 16 (31.4%) | 6 (23.1%) | 0.74 [0.33 ; 1.7] | 0.45 |
|  | RVPA conduit (*n=25)* | 8 (36.4%) | 1 (33.3%) | 0.92 [0.17 ; 5] | 0.92 |
